# Prediction of serosal invasion in gastric cancer: development and validation of multivariate models integrating preoperative clinicopathological features and radiographic findings based on late arterial phase CT images

**DOI:** 10.1186/s12885-021-08672-0

**Published:** 2021-09-16

**Authors:** Song Liu, Mengying Xu, Xiangmei Qiao, Changfeng Ji, Lin Li, Zhengyang Zhou

**Affiliations:** 1 Department of Radiology, Nanjing Drum Tower Hospital, The Affiliated Hospital of Nanjing University Medical School, No.321, Zhongshan Road, Nanjing City, 210008 Jiangsu Province China; 2 Department of Pathology, Nanjing Drum Tower Hospital, The Affiliated Hospital of Nanjing University Medical School, Nanjing, 210008 China

**Keywords:** Stomach neoplasms, Neoplasm staging, Tomography, X-ray computed, Endoscopy, Biomarkers, tumor

## Abstract

**Background:**

To develop and validate multivariate models integrating endoscopic biopsy, tumor markers, and CT findings based on late arterial phase (LAP) to predict serosal invasion in gastric cancer (GC).

**Methods:**

The preoperative differentiation degree, tumor markers, CT morphological characteristics, and CT value-related and texture parameters of 154 patients with GC were analyzed retrospectively. Multivariate models based on regression analysis and machine learning algorithms were performed to improve the diagnostic efficacy.

**Results:**

The differentiation degree, carbohydrate antigen (CA) 199, CA724, CA242, and multiple CT findings based on LAP differed significantly between T1–3 and T4 GCs in the primary cohort (all *P* < 0.05). Multivariate models based on regression analysis and random forest achieved AUCs of 0.849 and 0.865 in the primary cohort, respectively.

**Conclusion:**

We developed and validated multivariate models integrating endoscopic biopsy, tumor markers, CT morphological characteristics, and CT value-related and texture parameters to predict serosal invasion in GCs and achieved favorable performance.

**Supplementary Information:**

The online version contains supplementary material available at 10.1186/s12885-021-08672-0.

## Background

Gastric cancer (GC) is the fifth most common cancer and the third leading cause of cancer-related deaths globally and has become one of the major health burdens [1]. Previous studies have confirmed that serosal invasion is closely related to peritoneal seeding, which is generally regarded as a terrible condition [2, 3]. Thus, the precise preoperative prediction of serosal (visceral peritoneum) invasion is vital to select appropriate treatments and predict the outcome of GC. For instance, treatments such as staging laparoscopy and neoadjuvant chemotherapy would be scheduled in more detail if serosal (visceral peritoneum) invasion occurs [4, 5].

Both the seventh and eighth editions of the American Joint Cancer Committee divided the GC stage of T4 into T4a and T4b, adding the concept of T4a [6, 7]. T4a is defined as invading the serosa (in the anterior or posterior wall) or invading the visceral peritoneum (in the curvatures). Furthermore, the amount of subserosal fat tissue varies from person to person. Hence, the assessment of T4a varies depending on location and morphology [8–10]. It is difficult to predict T4a using only the morphological features of the tumor due to the above reasons. Therefore, making an accurate preoperative prediction of serosal invasion is quite challenging.

However, conventional computed tomography (CT) and endoscopic ultrasonography (EUS) are based on tumor morphology when evaluating the status of the serosa, which is inevitably limited by the above problems. Although EUS has certain advantages over CT in assessing T stage [11, 12], its results are obtained invasively and depend on operator experience without objective reflection on the overall staging (unable to accurately detect lymph nodes and distant metastases). Therefore, CT is still the most common staging tool for GC. In addition, studies on the quantitative analysis of GC using CT images have been widely carried out [13–17].

Recently, CT texture analysis that analyzes the distribution and relationship of pixel gray levels has developed rapidly [18]. Previous studies have confirmed that CT texture analysis and radiomics could predict T stages preoperatively in patients with advanced GC [13, 19, 20]. However, the postinjection delay of the arterial phase in these studies was 25–30 s. The major arteries can be clearly displayed in the early arterial phase (25–30 s), yet the mucosal layer may not be markedly enhanced simultaneously [21]. GC originates from the mucosal layer, so visualization of the mucosal lines is essential [22]. We assumed that the border of the gastric cancer would be displayed more clearly in the late arterial phase. In addition, various types of CT features, including morphology and CT values, can also be extracted [23, 24]. In clinical practice, a variety of clinicopathological information is collected prior to surgery, including endoscopic biopsy and multiple tumor markers. CT radiomics has been used to predict serosal invasion, but endoscopic biopsy and tumor markers were not included in the model [13]. Evaluating serosal invasion in GC is influenced by various factors. In recent years, GC staging has been predicted by integrating various types of preoperative clinicopathological information and radiographic findings [25, 26].

Thus, the purpose of our study was to develop and validate multivariate models integrating endoscopic biopsy, tumor markers, CT morphological characteristics, and CT value-related and texture parameters for predicting serosal invasion in GCs.

## Materials and methods

This retrospective study used deidentified data without protected health information, and it was approved by the Ethical Committee of Nanjing Drum Tower Hospital (Approval Documents Number: 2020–032-01). The requirement for informed consent was waived.

### Patients

Between April 2019 and April 2020, we searched the radiologic image archives of our hospital consecutively to identify 231 patients who had GC diagnosed by histopathologic analysis. The inclusion criteria were as follows: (1) pathological confirmation of GC postoperatively and (2) the availability of endoscopic biopsy, tumor markers, and abdominal contrast-enhanced CT within 2 weeks prior to surgery [25]. The exclusion criteria were as follows: (1) a history of GC treatment preoperatively (*n* = 8); (2) lacking 40 s LAP information (*n* = 24); (3) insufficient distention of the stomach (*n* = 18); (4) poor imaging quality due to respiratory or peristaltic motion (*n* = 7); (5) hardly visible due to the small size of the GC on CT images (long diameter < 1 cm) (*n* = 17); and (6) incomplete information on tumor markers (*n* = 3).

**Fig. 1:**
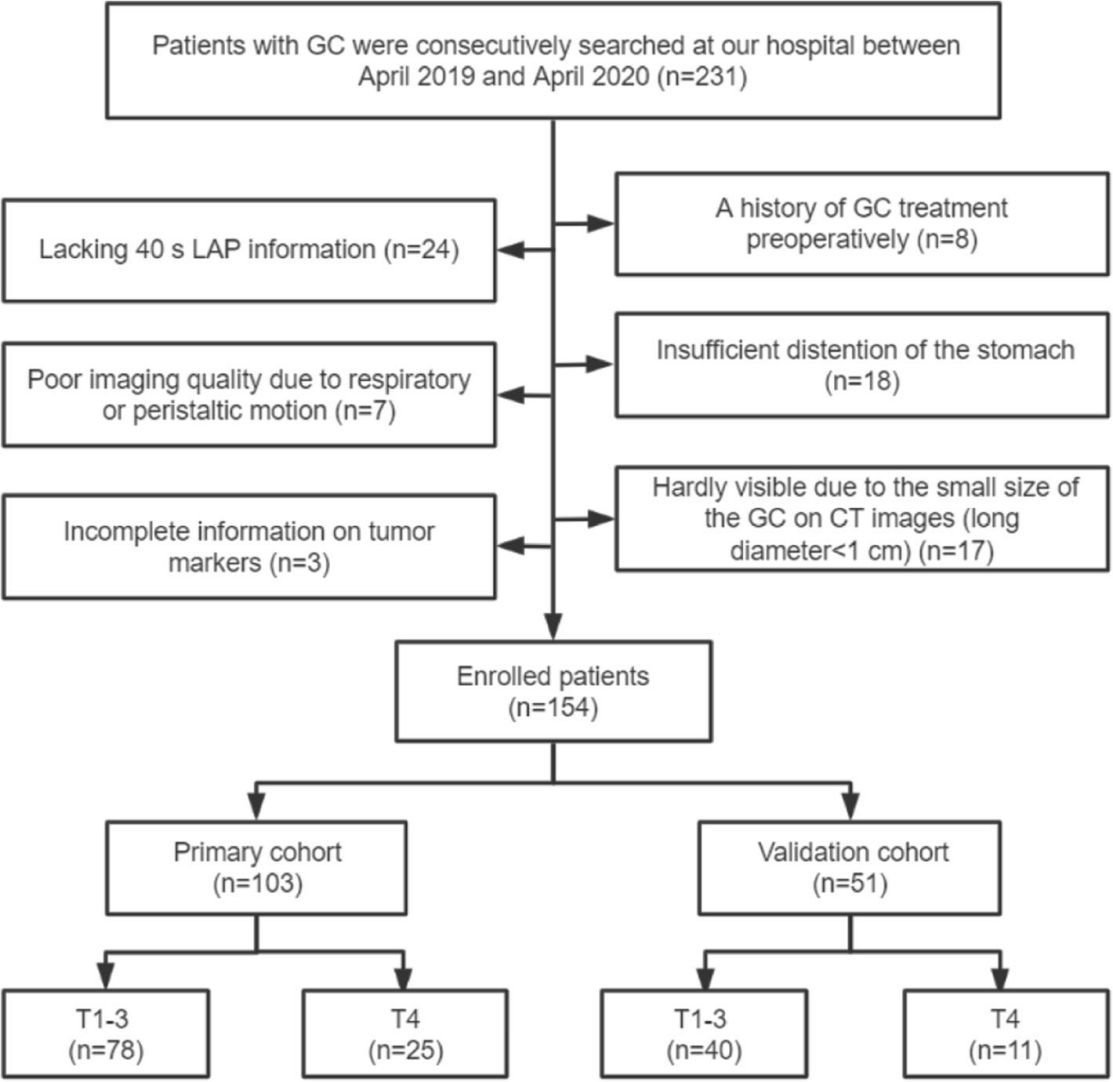
The flowchart of the patients enrolled in our study. GC, gastric cancer; LAP, late arterial phase; CT, computed tomography

**Fig. 2:**
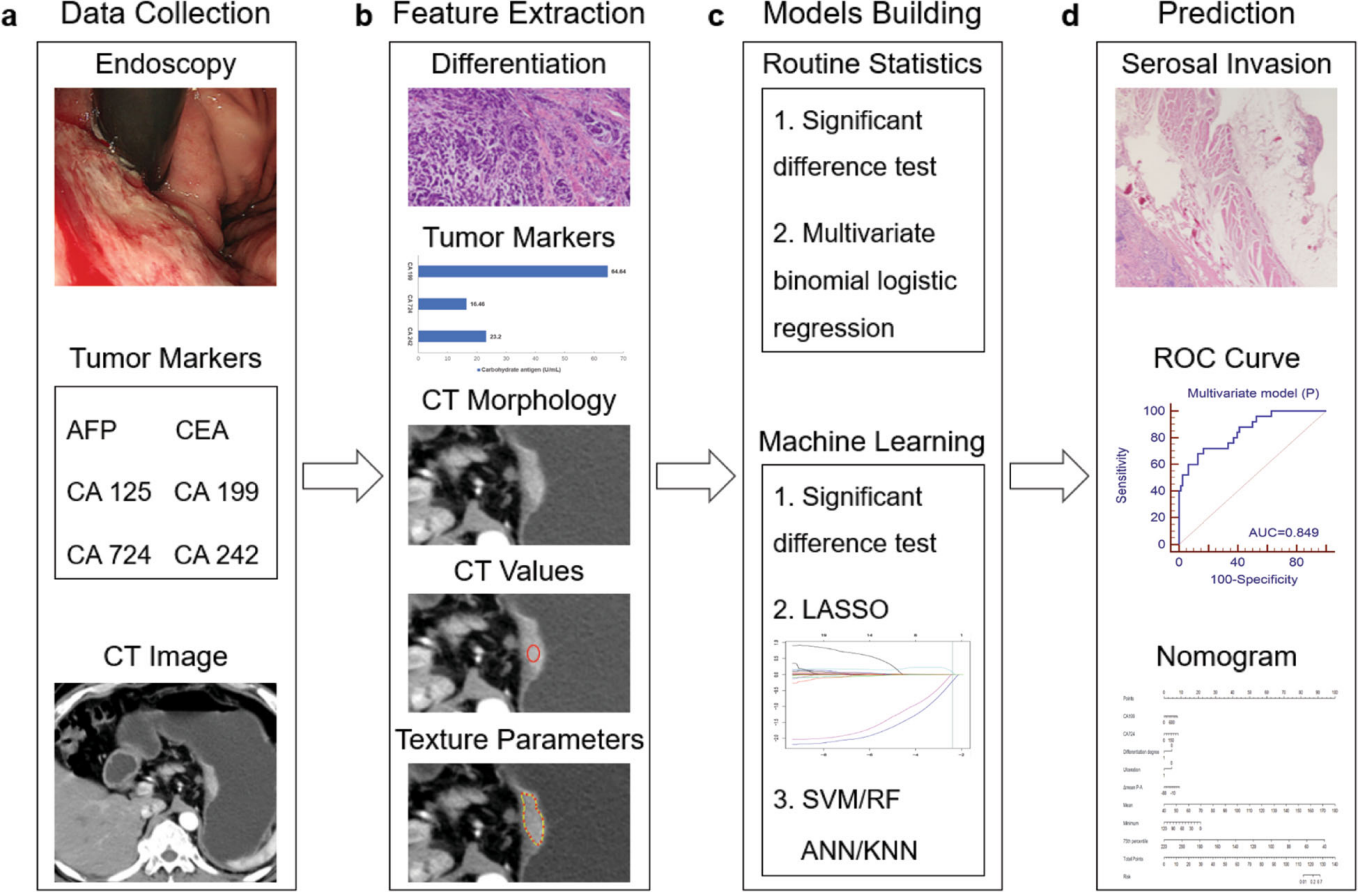
The workflow of this study. **a** Endoscopic biopsy, laboratory tests, and CT images of patients with gastric cancer were collected. **b** Differentiation degree based on biopsy, tumor markers, CT morphological characteristics based on late arterial phase, CT value-related parameters, and texture parameters were extracted. **c** Multivariate models were built based on binomial logistic regression and machine learning algorithms. **d** Diagnostic performance for predicting serosal invasion was obtained by ROC curve analysis, and a nomogram was used to visualize the multivariate model. AFP, alpha fetoprotein; CEA, carcinoembryonic antigen; CA, carbohydrate antigen; CT, computed tomography; LASSO, least absolute shrinkage and selection operator; SVM, support vector machine; RF, random forest; ANN, artificial neural network; KNN, k nearest neighbors; ROC, receiver operating characteristic

Ultimately, 154 patients (male, 107; female, 478; median age, 64 years; age range, 30–78 years) were included. The patients were divided into primary and validation cohorts based on the time of surgery at a ratio of 2:1. The flow chart of patient selection is shown in Fig. 1. The overall framework of this study is shown in Fig. 2. In addition, we added an extra cohort consisting of advanced GCs with negative tumor markers (83 patients).

### Endoscopic biopsy

Information on histological differentiation based on preoperative endoscopic biopsy was retrospectively examined and recorded by a pathologist (with 7 years of experience in pathological diagnosis of the digestive system) according to the WHO Classification of Tumors of the Digestive System (2019 version) [25, 27]. The tumors were classified into two groups: group 1, poor differentiation; group 2, moderate/well differentiation.

### Tumor markers

Six serum tumor markers, including alpha fetoprotein (AFP), carcinoembryonic antigen (CEA), carbohydrate antigen (CA) 125, CA199, CA724, and CA242, were collected within 2 weeks before surgery [25].

### CT image acquisition

CT examinations were performed on a 64-row scanner (uCT 780, United Imaging, Shanghai, China). All patients were required to fast for at least 6 h and take 600–1000 mL of warm water orally prior to the examination. All patients were placed in the supine position, and the scan covered the upper or entire abdomen. Following the nonenhanced scan, 1.5 mL/kg iodinated contrast agent (Omnipaque 350 mg I/mL, GE Healthcare) was injected intravenously at a flow rate of 3.0 mL/s using a high-pressure syringe. Imaging was achieved with postinjection delays of 40, 70, and 180 s after the initiation of contrast material injection, corresponding to the late arterial, portal, and delayed phases, respectively. The CT scan parameters were as follows: tube voltage 120 kV, tube current 150–250 mA, field of view 35–50 cm, matrix 512 × 512, rotation time 0.7 s, and pitch 1.0875. The images were reconstructed with section thicknesses of 1 and 5 mm; the former were used for multiplanar reconstruction, and the latter were used for the measurement of CT values due to the signal-to-noise ratio [25].

### Image analysis

#### Morphological characteristics

Readers 1 and 2 (with 5 and 7 years of experience in abdominal imaging, respectively), who were blinded to the clinicopathological information of the patients except for the general location of the tumors, independently evaluated the morphological characteristics of each lesion on the 40 s LAP CT images, and their results were used to assess interobserver agreement. Any discrepant opinions between readers 1 and 2 were resolved by reader 3 (with 20 years of experience in abdominal imaging) as the final result [25]. The characteristics were: 1) major location (cardia, body, and antrum); 2) tumor range (1 location, ≥2 locations); 3) major orientation (lesser curvature, greater curvature, anterior wall, and posterior wall); 4) circumferential range (1/4, 2/4, 3/4, and 4/4); 5) infiltrative growth (absent, present): unclear border between the lesion and the normal gastric wall; 6) ulceration (absent, present); 7) adjacent adipose tissue stains (absent, present); 8) mucosal line status (interruption, thickening); 9) morphological type (thickening type, mass type); 10) linitis plastica (absent, present); and 11) lymphadenectasis (absent, present): the short axis of the regional lymph node was greater than 1 cm.

#### CT value-related parameters

The oval regions of interest (ROIs) were drawn to encompass the area of greatest enhancement on the maximal section in 40 s LAP and were copied on the same slice in the other three phases by reader 1. The mean size of the ROIs was 32.82 mm^2^, and the range was 6.60–156.90 mm^2^. The mean CT attenuation values of the tumor in the nonenhanced, late arterial, portal, and delayed phases were recorded as the N value mean, AP value mean, PP value mean, and DP value mean, respectively, as well as the maximum and minimum CT values. With the N, AP, and PP value means as the references, postcontrast tumorous attenuation differences (Δmean A-N, Δmean P-N, Δmean D-N, Δmean P-A, Δmean D-A, and Δmean D-P) were calculated. CT value-related parameters derived from the ROIs delineated by reader 1 were used to predict serosal invasion. To determine the interobserver reproducibility, reader 2 repeated the above procedure [25].

#### CT texture analysis

**Fig. 3:**
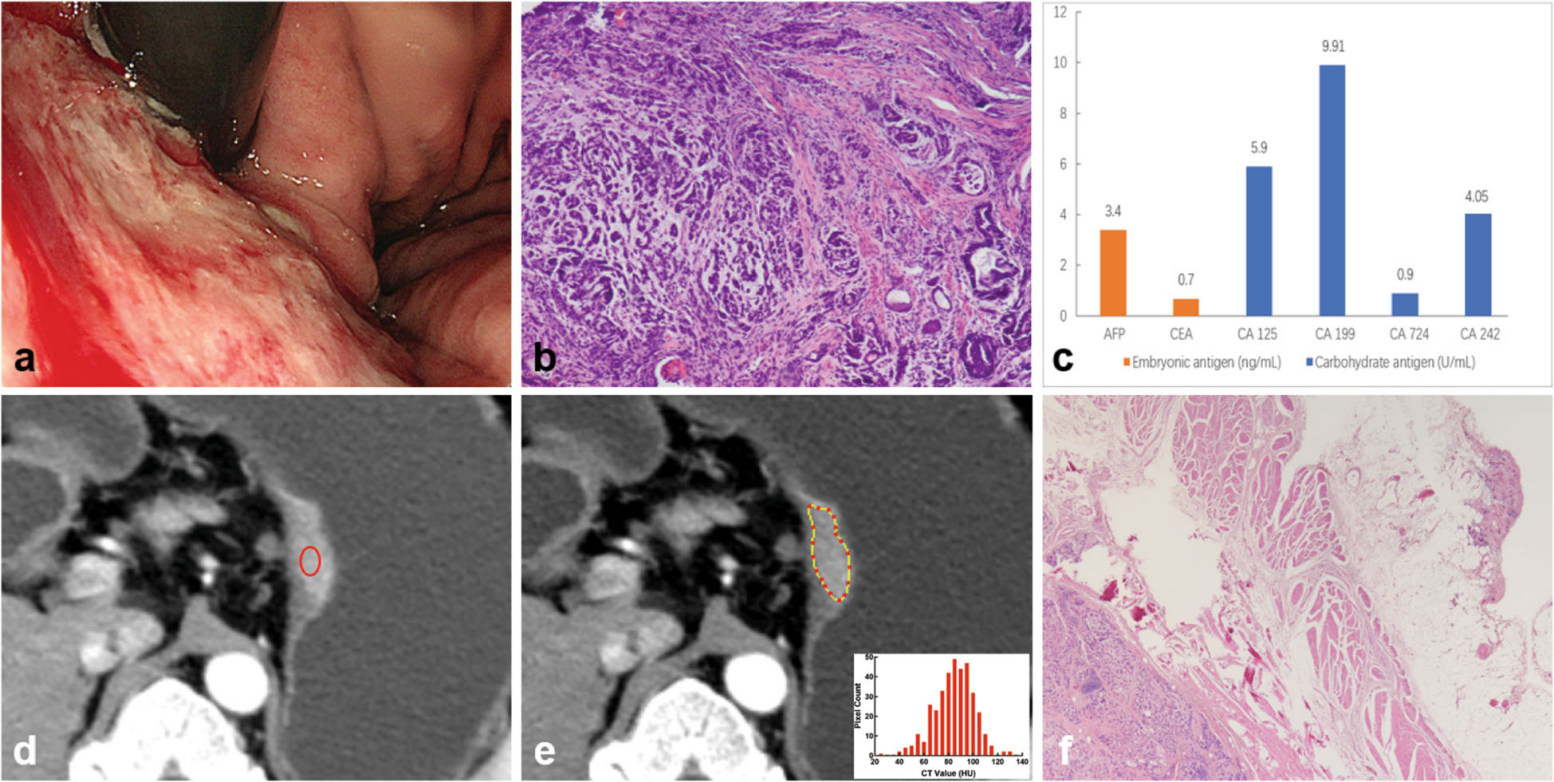
A 50-year-old man with gastric cancer pathologically diagnosed with serosal invasion. **a** The endoscopic image indicates a mass lesion in the posterior wall of the stomach body. **b** Hematoxylin and eosin (H&E) staining of a specimen based on endoscopic biopsy shows a poorly differentiated carcinoma (original magnification, × 100). **c** The values of the tumor markers, including AFP, CEA, CA125, CA199, CA724, and CA242, were 3.40 ng/mL, 0.70 ng/mL, 5.90 U/mL, 9.91 U/mL, 0.90 U/mL, and 4.05 U/mL, respectively. **d** The late arterial phase computed tomography (CT) image shows a mass lesion with marked enhancement in the posterior wall of the stomach body. An oval region of interest (ROI) was drawn to encompass the area of greatest enhancement on the maximal section, and the CT value-related parameters were extracted. **e** A polygonal ROI was manually drawn along the margin of the tumor on the largest cross-section, and the texture parameters were extracted. **f** H&E staining of a postoperative specimen confirms gastric cancer with serosal invasion (original magnification, × 20). AFP, alpha fetoprotein; CEA, carcinoembryonic antigen; CA, carbohydrate antigen

The LAP CT images were uploaded into in-house software (Image Analyzer 2.0, China). All the images were reviewed by reader 1. Polygonal ROIs (mean size, 402.23 mm^2^; range, 24.36–2442.87 mm^2^) were manually drawn along the margin of the tumor on the largest cross-section (Fig. 3), avoiding the normal gastric wall tissue and the gastric cavity contents. Texture parameters were as follows: (1) the first-order features included the mean, standard deviation, max frequency, mode, minimum, maximum, cumulative percentiles (the 5th, 10th, 25th, 50th, 75th, and 90th percentiles), skewness, kurtosis, entropy, and histogram width; (2) the second-order features were from the gray-level cooccurrence matrix (GLCM) and included Entropy GLCM, Energy GLCM, Inertia GLCM, and Variance GLCM. Texture parameters derived from the ROIs delineated by reader 1 were used to predict serosal invasion. To determine the interobserver reproducibility, reader 2 repeated the above procedure [25].

### Development, performance, and validation of the multivariate models

Significant (*P* < 0.05) variables in the univariate analysis were input into a multivariate binomial logistic regression based on a backward elimination process in the primary cohort. The Hosmer-Lemeshow test was used to measure the goodness of fit. A nomogram was constructed based on the multivariate model in the primary cohort with the R software package (version 3.5.2: http://www.Rproject.org). The multivariate model was applied to the validation cohort and the extra cohort. The diagnostic efficacy was evaluated with receiver operating characteristic (ROC) curve analysis [25].

If significance (*P* < 0.05) was met in the univariate analysis of the primary cohort, the variables were incorporated into the least absolute shrinkage and selection operator (LASSO) for dimension reduction. Then, we built four machine learning models, including support vector machine (SVM), random forest (RF), artificial neural network (ANN) and k nearest neighbors (KNN), with the LASSO selected features as the input factors. The fivefold cross-validation was performed to improve and compare these models’ performances. The best performing model was selected to build the predictive model for serosal invasion and applied to the validation cohort [25, 28].

### Pathological assessment after surgery

All patients underwent gastrectomy (either total or partial). All gastric specimens were processed according to standard pathological procedures. The pathological T stage was retrospectively examined and recorded according to the 8th American Joint Committee on Cancer classification [7, 25]. The patients were divided into two groups (T1–3 vs. T4).

### Statistical analysis

The differences in demographic data, endoscopic biopsy, and morphological characteristics were assessed with the chi-square or Fisher’s exact test (*n* < 5). Kappa statistics were applied to evaluate the interobserver consistency. A kappa value of less than 0.200 was considered poor, 0.201–0.400 was considered fair, 0.401–0.600 was considered moderate, 0.601–0.800 was considered good, and 0.801–1.000 was considered excellent. The normality distributions of the tumor markers, CT value-related parameters, and texture parameters were evaluated by the Shapiro-Wilk test. Based on the normality test results, the differences between T1–3 and T4 were analyzed by the Mann-Whitney U test. ROC curve analysis was performed, and the area under the ROC curve (AUC), diagnostic sensitivity, specificity, and accuracy were calculated. The cutoff value was established by calculating the largest Youden index (Youden index = sensitivity+specificity-1). The interobserver agreement of the CT value-related and texture parameters was estimated with the intraclass correlation coefficient (ICC) (0.000–0.200: poor; 0.201–0.400: fair; 0.401–0.600: moderate; 0.601–0.800: good; 0.801–1.000: excellent). All statistical analyses were performed with SPSS (version 22.0 for Microsoft Windows × 64, SPSS), MedCalc Statistical Software (version 11.4.2.0 MedCalc Software bvba; http://www.medcalc.org; 2011), and R software (version 3.5.2: http://www.Rproject.org). A two-tailed *P* value< 0.05 was considered statistically significant [25].

## Results

### Qualitative analysis

Table 1 summarizes the results of the univariate analysis of the demographic data, endoscopic biopsy, and morphological characteristics between the T1–3 and T4 groups in the primary and validation cohorts. The differentiation degree, infiltrative growth, ulceration, and morphological type differed significantly between the two groups in the primary cohort (all *P* < 0.05). There were no significant differences in major location, tumor range, major orientation, circumferential range, adjacent adipose tissue stains, mucosal line status, linitis plastica, or lymphadenectasis between the two groups in the primary cohort (all *P* > 0.05). There were significant differences for the six characteristics in the validation cohort (all *P* < 0.05).

**Table 1:** Univariate analysis of demographic data, endoscopic biopsy, and morphological characteristics in the primary and validation cohorts

Characteristics	Primary cohort		*P* value	Validation cohort		*P* value
	T1–3 (*n* = 78)	T4 (*n* = 25)		T1–3 (*n* = 40)	T4 (*n* = 11)	
Demographic data						
Gender			0.154			0.214
Male	58	22		23	4	
Female	20	3		17	7	
Age (y)			0.543			1.000
< 60	29	11		13	3	
≥ 60	49	14		27	8	
Endoscopic biopsy						
Differentiation degree			0.004*			0.141
Poor	48	23		26	10	
Moderate & Well	30	2		14	1	
Morphological characteristics						
Major location			0.884			0.690
Cardia	21	6		14	2	
Body	27	8		14	5	
Antrum	30	11		12	4	
Tumor range			0.121			0.009*
1 location	51	12		32	4	
≥ 2 locations	27	13		8	7	
Major orientation			0.637			0.591
Lesser curvature	35	11		19	7	
Greater curvature	2	2		3	0	
Anterior wall	7	2		2	1	
Posterior wall	34	10		16	3	
Circumferential range			0.428			0.010*
1/4	34	7		24	1	
2/4	26	11		10	7	
3/4	11	3		3	1	
4/4	7	4		3	2	
Infiltrative growth			0.009*			0.001*
Absent	54	10		30	2	
Present	24	15		10	9	
Ulceration			0.036*			0.305
Absent	20	12		20	8	
Present	58	13		20	3	
Adjacent adipose tissue stains			0.267			0.009*
Absent	56	15		25	2	
Present	22	10		15	9	
Mucosal line status			0.280			0.002*
Interruption	28	12		11	9	
Thickening	50	13		29	2	
Morphological type			0.026*			0.037*
Thickening type	12	9		11	7	
Mass type	66	16		29	4	
Linitis plastica			0.091			0.292
Absent	76	22		37	9	
Present	2	3		3	2	
Lymphadenectasis			1.000			0.598
Absent	66	21		36	9	
Present	12	4		4	2	

*P < 0.05 with chi-square test or Fisher’s exact test (*n* < 5)

### Quantitative analysis

#### Tumor markers

The values of CA199, CA724, and CA242 differed significantly in the primary cohort (*P* = 0.007, 0.023, and 0.015, respectively, Table 2). There were no significant differences in CEA, CA125, or AFP between the T1–3 and T4 groups in the primary cohort (all *P* > 0.05, Table A1).

**Table 2:** Statistical description and univariate analysis of tumor markers, the CT value-related parameters, and texture parameters in the primary cohort

Parameters	T1–3	T4	*P* value
Tumor markers			
CA199 (U/mL)	8.92 (5.55, 12.91)	11.74 (8.39, 197.35)	0.007*
CA724 (U/mL)	1.83 (1.20, 4.69)	3.00 (1.73, 22.22)	0.023*
CA242 (U/mL)	3.19 (2.21, 5.54)	6.91 (2.69, 105.51)	0.015*
CT value-related parameters			
AP value mean (HU)	112.51 (89.22, 137.63)	94.12 (76.98, 113.02)	0.024*
AP value min (HU)	90.00 (71.50, 113.00)	70.00 (54.50, 101.50)	0.018*
Δmean A-N (HU)	69.86 (50.52, 96.24)	50.28 (34.13, 75.73)	0.028*
Δmean P-A (HU)	−19.02 (−35.97, −6.23)	−6.12 (−24.34, 7.84)	0.024*
Δmean D-A (HU)	−32.65 (−50.80, −10.62)	−17.50 (−36.10, 14.62)	0.021*
Texture parameters			
Mean (HU)	106.47 (92.20, 120.32)	90.74 (71.18, 111.48)	0.027*
Max frequency	15.50 (10.00, 29.00)	31.00 (19.00, 51.00)	< 0.001*
Minimum (HU)	58.00 (41.75, 75.25)	42.00 (25.50, 55.00)	0.006*
5th percentile (HU)	76.50 (63.50, 93.00)	63.00 (45.00, 79.50)	0.015*
10th percentile (HU)	82.00 (68.00, 101.00)	68.00 (51.00, 89.00)	0.019*
25th percentile (HU)	93.00 (79.75, 111.00)	78.00 (59.50, 98.50)	0.017*
50th percentile (HU)	106.00 (91.75, 120.50)	90.00 (68.50, 109.50)	0.029*
75th percentile (HU)	118.50 (103.00, 133.25)	101.00 (84.50, 123.50)	0.027*
90th percentile (HU)	129.00 (114.75, 147.00)	114.00 (95.50, 141.00)	0.046*

The data are presented as median with (1st quartile, 3rd quartile)

*CA* carbohydrate antigen, *AP* arterial phase

*P < 0.05 with Mann-Whitney U test

#### CT value-related and texture parameters

The results of the univariate analysis for quantitative CT value-related and texture parameters between the T1–3 and T4 groups in the primary cohort are shown in Table 2. For the CT value-related parameters, there were significant differences in AP value mean, AP value min, Δmean A-N, Δmean P-A, and Δmean D-A (all *P* < 0.05). For the texture parameters, nine parameters differed significantly between the two groups (all *P* < 0.05), and the corresponding AUCs ranged from 0.633 to 0.742 (Table 3).

**Table 3:** The diagnostic performance of tumor markers, the CT value-related parameters, and texture parameters in the primary cohort

Parameters	Cutoff	Sensitivity	Specificity	AUC	Accuracy	*P* value
Tumor markers						
CA199 (U/mL)	9.47	0.720	0.615	0.681	0.640	0.009*
CA724 (U/mL)	1.48	0.840	0.436	0.652	0.534	0.022*
CA242 (U/mL)	6.30	0.520	0.821	0.662	0.748	0.021*
CT value-related parameters						
AP value mean (HU)	96.62	0.680	0.692	0.651	0.689	0.031*
AP value min (HU)	78.00	0.680	0.680	0.658	0.680	0.024*
Δmean A-N (HU)	54.11	0.640	0.705	0.647	0.689	0.035*
Δmean P-A (HU)	−7.46	0.560	0.744	0.650	0.699	0.025*
Δmean D-A (HU)	−25.09	0.680	0.603	0.654	0.622	0.021*
Texture parameters						
Mean (HU)	90.74	0.520	0.782	0.648	0.718	0.036*
Max frequency	20.00	0.760	0.680	0.742	0.699	< 0.001*
Minimum (HU)	56.00	0.800	0.539	0.682	0.602	0.005*
5th percentile (HU)	69.00	0.640	0.680	0.662	0.670	0.014*
10th percentile (HU)	70.00	0.560	0.744	0.657	0.699	0.021*
25th percentile (HU)	89.00	0.720	0.603	0.659	0.631	0.021*
50th percentile (HU)	90.00	0.520	0.769	0.645	0.709	0.038*
75th percentile (HU)	101.00	0.520	0.782	0.647	0.718	0.038*
90th percentile (HU)	107.00	0.440	0.846	0.633	0.747	0.064

*AUC* area under the receiver operating characteristic (ROC) curve, *CA* carbohydrate antigen, *AP* arterial phase

*P < 0.05 with ROC curve analysis

### Development, performance, and validation of the multivariate models

#### Multivariate binomial logistic regression

**Fig. 4:**
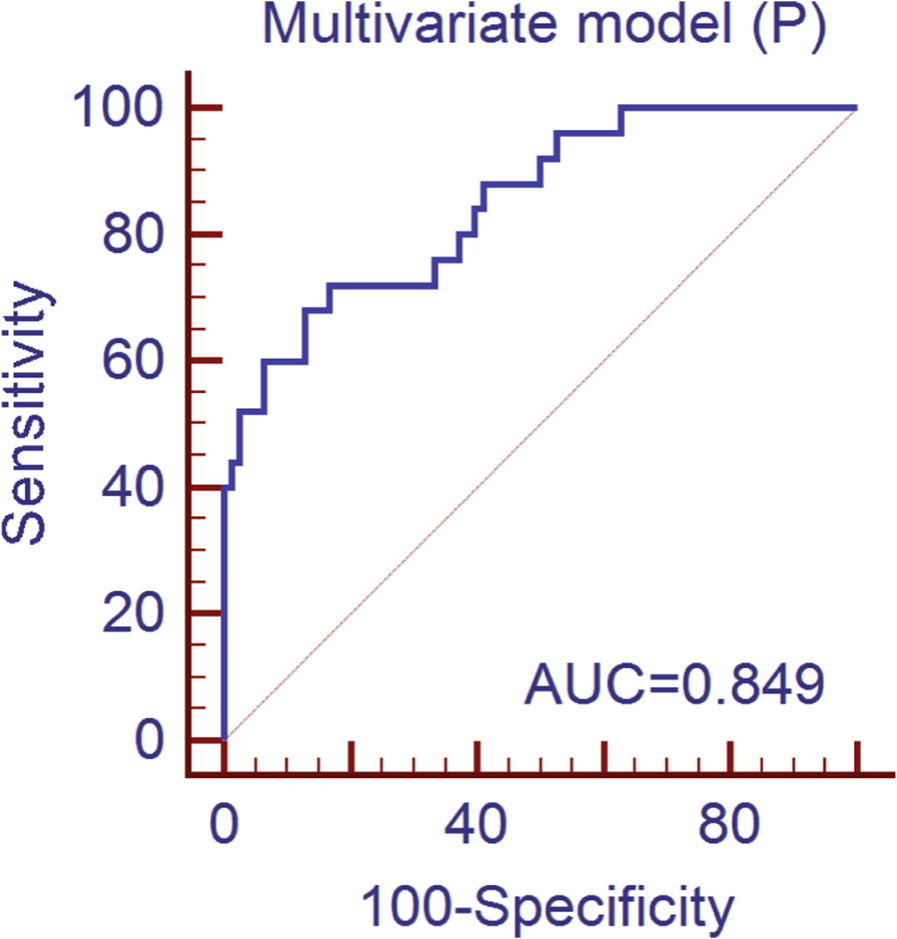
The receiver operating characteristic (ROC) curve of the multivariate model based on binomial logistic regression analysis for predicting the serosal invasion of gastric cancer in the primary cohort. The AUC of the multivariate model was 0.849. AUC, area under the receiver operating characteristic curve

**Fig. 5:**
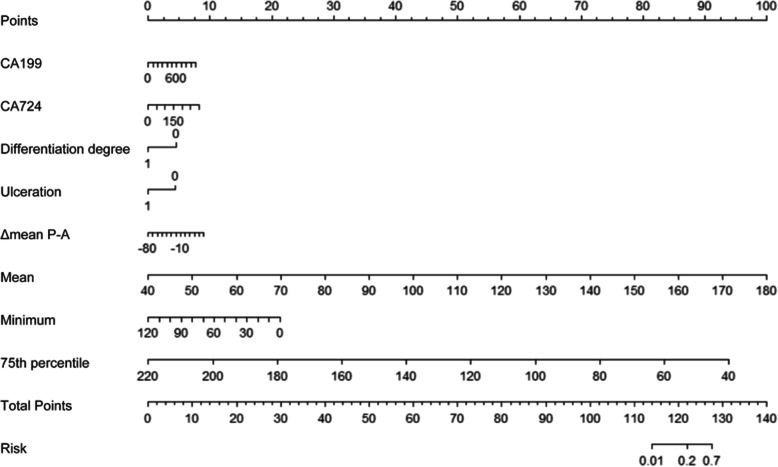
A nomogram based on a multivariate logistic regression model for predicting the serosal invasion of gastric cancer in the primary cohort using the variables of CA199, CA724, differentiation degree, ulceration, Δmean P-A, mean, minimum, and 75th percentile. CA, carbohydrate antigen

The best-performing model based on regression for predicting serosal invasion in the primary cohort consisted of differentiation degree, ulceration, CA199, CA724, Δmean P-A, mean, minimum, and 75th percentile. The multivariate model had a predictive ability with a cutoff of 0.24 (AUC = 0.849, *P* < 0.001), which yield a sensitivity, specificity, and accuracy of 72.0, 83.3, and 80.6%, respectively. The ROC curve of the primary cohort is plotted in Fig. 4. The cutoff value of 0.24 was used to test the predictive performance of the validation cohort, which yield a sensitivity, specificity, and accuracy of 81.8, 82.5, and 82.4%, respectively. A nomogram constructed based on the multivariate logistic regression model in the primary cohort for predicting serosal invasion is displayed in Fig. 5.

In addition, the cutoff value of 0.24 was used to test the predictive performance of the extra cohort consisting of advanced GCs with negative tumor markers (83 patients), which yield a sensitivity, specificity, and accuracy of 53.3, 76.5, and 72.3%, respectively.

#### Machine learning algorithms

**Fig. 6:**
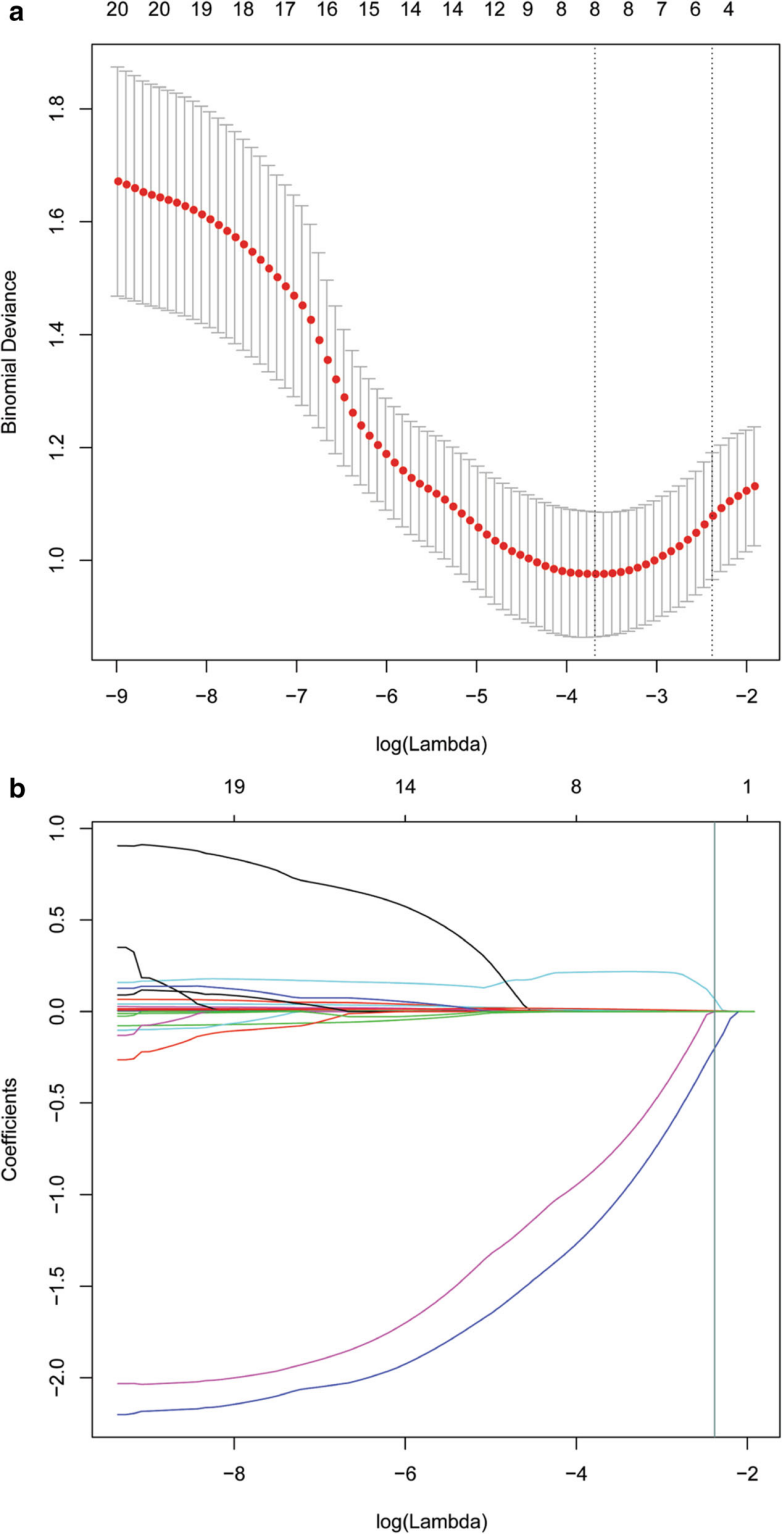
Feature selection was performed using the least absolute shrinkage and selection operator (LASSO) regression model. **a** Tuning parameter (λ) selection in the LASSO model used fivefold cross-validation via minimum criteria. Vertical lines were drawn at the optimal values using the minimum criteria and 1 standard error of the minimum criteria. The optimal λ value of 0.0922 with log (λ) = − 2.3838 was chosen. **b** LASSO coefficient profiles of the 21 selected features. A coefficient profile plot was generated versus the selected log (λ) value using fivefold cross-validation; five selected features with nonzero coefficients were retained

LASSO was applied to reduce the dimensions and to select optimal variables in the primary cohort (Fig. 6). Finally, infiltrative growth, ulceration, CA242, CA724, and minimum were integrated to build multivariate models using the SVM, RF, ANN, and KNN algorithms. The multivariate model generated by RF showed best performance in the four machine learning algorithms with an AUC of 0.865. The developed model based on RF was also applied in the validation cohort and achieved an AUC of 0.845.

### Interobserver agreement

All CT morphological characteristics showed good to excellent interobserver agreement in the evaluation of GCs (κ = 0.715–0.902) (Table A2). All CT value-related parameters (ICC = 0.687–0.941) and texture parameters (ICC = 0.706–0.989) also showed good to excellent interobserver agreement (Tables A3 and A4).

## Discussion

In the current study, we investigated the ability of multivariate models integrating preoperative clinicopathological features and radiographic findings based on LAP CT images to predict serosal invasion in GC. To build the multivariate models, the differentiation degree based on endoscopic biopsy, 6 tumor markers, 11 CT morphological characteristics, 18 CT value-related parameters, and 32 CT texture parameters were collected. There were significant differences in multiple features between the T1–3 and T4 groups.

Endoscopic biopsy and tumor markers are widely used in the early diagnosis and disease monitoring in gastric cancer [5, 29, 30]. In this study, we demonstrated that the differentiation degree based on biopsy, CA199, CA724, and CA242 were able to predict serosal invasion in GC. Poorly differentiated GC is usually more aggressive and carries a higher risk of deeper invasion. Kim DK et al. reported that the deep submucosal invasion of GC was related to the poorly differentiated type [31]. This was consistent with our results. Additionally, the tumor markers indirectly reflect the changes in related gene expression during tumor progression. A previous study reported that preoperative serum CA 242 values can serve as an independent prognostic marker for GC patients [30].

We found that the morphological characteristics in 40s LAP, including infiltrative growth, ulceration, and morphological type, differed significantly between the T1–3 and T4 groups in the primary cohort. When accompanied by infiltrative growth, ulceration, and thickening type, GC is more aggressive and more likely to invade the serosa. However, there were significant differences in six characteristics in the validation cohort. The different results of the morphological characteristics analysis in the primary and validation cohorts could be explained by the different sample sizes. The primary cohort had a larger sample size and thus might be more representative.

In quantitative analysis, we used not only CT value-related parameters but also texture parameters. For the CT value-related parameters, there were significant differences in AP value mean, AP value min, Δmean A-N, Δmean P-A, and Δmean D-A between the T1–3 and T4 groups in the primary cohort. This indicates that the parameters related to the LAP may better reflect tumor information. Furthermore, nine texture parameters, including the mean, max frequency, minimum, and 5th–90th percentiles, differed significantly in the primary cohort. GCs with serosal invasion had a higher max frequency value (indicating the peak value of the histogram). The 5th–90th percentiles reflected the enhancement degree of different components of the tumor. GCs with serosal invasion tend to be more aggressive and grow rapidly, resulting in insufficient blood supply and necrosis [32].

To develop multivariate models for predicting serosal invasion in GC, the regression analysis and machine learning algorithms were used in the study. For the regression model, we utilized a backward elimination process [33]. For the machine learning algorithm, LASSO was used for dimension reduction, which is in general use [13, 17, 34]. The multivariate models based on regression analysis and the RF algorithm showed satisfactory performance in the primary cohort (AUC = 0.849 and 0.865, respectively). The AUC of multivariate model generated by RF was slightly better than regression analysis. Furthermore, we applied the Delong test to compare the performance of the models based on RF and regression analysis and found that there was no significant difference between the two models. The developed models were also used in the validation cohort and achieved better performance.

In addition, the cutoff value of 0.24 (the same as the regression model developed in the primary cohort) was used to test the predictive performance of the extra cohort consisting of advanced GCs with negative tumor markers (83 patients), which yield an accuracy of 72.3%.

Most previous works applied to predict serosal invasion in GC focused on morphology [8–10, 35]. Although the number of studies was abundant, the results varied widely and were controversial. In recent years, CT radiomics has been used to predict serosal invasion, while endoscopic biopsy and tumor markers were not included in the model building [13]. Evaluating serosal invasion in GC is influenced by various factors. In this situation, to solve the above problems, a comprehensive evaluation based on preoperative clinicopathological features and radiographic findings was adopted, and it achieved a satisfactory result. It is worth mentioning that the interobserver agreement for all morphological characteristics, CT value-related parameters, and texture features proved to be good or excellent.

There are some limitations of this study. First, it used retrospective data collected from a single center, and the sample might be biased. Second, the texture features were derived from the two-dimensional ROIs of lesions with manual segmentation, which might have lost feature information in the longitudinal direction. Third, only the biopsy information was extracted, and more detailed information needs to be included from endoscopy. Thus, additional investigation to remedy the abovementioned insufficiencies should be performed.

In conclusion, we developed and validated multivariate models integrating preoperative clinicopathological features and radiographic findings based on LAP CT images to predict serosal invasion in GCs, and it achieved a favorable performance.

## Supplementary Information

**Additional file 1: Table A1.** Statistical description and univariate analysis of tumor markers, the CT value-related parameters, and texture parameters in the primary cohort (*P* > 0.05). **Table A2.** Interobserver agreement for CT morphological characteristics. **Table A3.** Interobserver agreement for CT value-related parameters. **Table A4.** Interobserver agreement for texture parameters.

## Data Availability

The datasets used and analyzed during the current study are available from the corresponding author on reasonable request.

## Abbreviations

AFP: Alpha fetoprotein
ANN: Artificial neural network
AP: Arterial phase
AUC: Area under the curve
CA: Carbohydrate antigen
CEA: Carcinoembryonic antigen
CT: Computed tomography
DP: Delayed phase
EUS: Endoscopic ultrasonography
GC: Gastric cancer
GLCM: Gray-level cooccurrence matrix
ICC: Intraclass correlation coefficient
KNN: K nearest neighbors
LAP: Late arterial phase
LASSO: Least absolute shrinkage and selection operator
N: Non-enhanced
PP: Portal phase
RF: Random forest
ROC: Receiver operating characteristic
ROIs: Regions of interest
SVM: Support vector machine
