# Supplementary material for: Prediction of serosal invasion in gastric cancer: development and validation of multivariate models integrating preoperative clinicopathological features and radiographic findings based on late arterial phase CT images
Source: BMC Cancer. 2021 Sep 16;21:1038. doi: 10.1186/s12885-021-08672-0 (PMC8447770; doi:10.1186/s12885-021-08672-0)
Supplement: Supplementary file 1 — Additional file 1: Table A1. Statistical description and univariate analysis of tumor markers, the CT value-related parameters, and texture parameters in the primary cohort (P > 0.05). Table A2. Interobserver agreement for CT morphological characteristics. Table A3. Interobserver agreement for CT value-related parameters. Table A4. Interobserver agreement for texture parameters. [file 12885_2021_8672_MOESM1_ESM.docx]

**Table A1** Statistical description and univariate analysis of tumor markers, the CT value-related parameters, and texture parameters in the primary cohort (*P*>0.05)

| Parameters | T1-3 | T4 | *P* value |
| --- | --- | --- | --- |
| Tumor markers | | | |
| AFP (ng/mL) | 2.00 (1.40, 3.63) | 2.80 (1.60, 3.80) | 0.378 |
| CEA (ng/mL) | 1.42 (0.70, 3.12) | 1.85 (0.97, 5.42) | 0.161 |
| CA125 (U/mL) | 7.90 (5.10, 10.95) | 7.90 (6.80, 10.25) | 0.294 |
| CT value-related parameters | | | |
| N value mean (HU) | 41.48 (38.06, 44.93) | 41.16 (34.75, 45.18) | 0.593 |
| N value max (HU) | 54.00 (50.00, 61.00) | 55.00 (49.50, 61.00) | 0.972 |
| N value min (HU) | 28.00 (21.00, 34.00) | 27.00 (18.00, 30.00) | 0.266 |
| AP value max (HU) | 128.00 (110.00, 159.50) | 118.00 (97.00, 127.50) | 0.062 |
| PP value mean (HU) | 90.12 (80.27, 105.71) | 91.38 (78.54, 99.86) | 0.732 |
| PP value max (HU) | 110.00 (98.50, 123.00) | 110.00 (100.50, 122.00) | 0.881 |
| PP value min (HU) | 71.50 (60.00, 86.00) | 71.00 (55.00, 81.00) | 0.374 |
| DP value mean (HU) | 81.48 (72.51, 91.95) | 80.65 (73.04, 97.55) | 0.695 |
| DP value max (HU) | 99.50 (87.00, 110.25) | 107.00 (93.00, 122.00) | 0.217 |
| DP value min (HU) | 63.50 (53.00, 74.00) | 66.00 (53.00, 80.00) | 0.823 |
| Δmean P-N (HU) | ~~6~~48.96 (38.62, 64.54) | 49.52 (41.41, 57.37) | 0.884 |
| Δmean D-N (HU) | 40.38 (28.93, 51.22) | 45.82 (31.11, 56.88) | 0.531 |
| Δmean D-P (HU) | -8.57 (-21.49, -0.07) | -5.64 (-11.08, 6.08) | 0.173 |
| Texture parameters | | | |
| Standard deviation | 17.78 (16.64, 22.03) | 17.90 (15.09, 20.91) | 0.601 |
| Mode (HU) | 102.00 (85.00, 123.00) | 92.00 (69.50, 114.00) | 0.097 |
| Maximum (HU) | 155.50 (140.75, 172.75) | 147.00 (132.50, 171.50) | 0.288 |
| Skewness | 0.04 (-0.14, 0.28) | 0.22 (-0.15, 0.43) | 0.162 |
| Kurtosis | 2.86 (2.47, 3.23) | 2.94 (2.54, 3.48) | 0.196 |
| Entropy | 4.11 (3.93, 4.34) | 4.20 (3.98, 4.33) | 0.415 |
| Histogram width (HU) | 46.00 (37.00, 58.25) | 42.00 (37.50, 55.50) | 0.518 |
| Entropy GLCM 10 | 7.13 (6.79, 7.58) | 7.45 (6.97, 7.55) | 0.142 |
| Entropy GLCM 11 | 6.95 (6.60, 7.39) | 7.18 (6.81, 7.33) | 0.258 |
| Entropy GLCM 12 | 7.09 (6.75, 7.57) | 7.35 (7.03, 7.63) | 0.155 |
| Entropy GLCM 13 | 6.85 (6.60, 7.35) | 7.08 (6.81, 7.35) | 0.196 |
| Energy GLCM 10 ^a^ | 9.48 (6.71, 11.22) | 8.~~28~~03 (6.88, 10.86) | 0.292 |
| Energy GLCM 11 ^a^ | 10.55 (7.82, 13.49) | 9.84 (7.96, 12.22) | 0.474 |
| Energy GLCM 12 ^a^ | 9.70 (6.89, 11.84) | 8.50 (6.69, 11.03) | 0.271 |
| Energy GLCM 13 ^a^ | 11.31 (8.31, 13.43) | 10.27 (8.07, 12.29) | 0.397 |
| Inertia GLCM 10 | 9.78 (8.08, 13.19) | 8.73 (7.20, 10.51) | 0.071 |
| Inertia GLCM 11 | 6.30 (5.22, 8.07) | 5.93 (4.87, 6.79) | 0.082 |
| Inertia GLCM 12 | 9.65 (7.63, 12.51) | 8.69 (7.41, 10.84) | 0.176 |
| Inertia GLCM 13 | 5.76 (4.64, 7.51) | 5.13 (4.42, 6.40) | 0.186 |
| Variance GLCM 10 | 19.11 (12.50, 29.05) | 18.68 (13.30, 25.23) | 0.689 |
| Variance GLCM 11 | 19.65 (12.65, 29.42) | 18.67 (13.71, 26.64) | 0.678 |
| Variance GLCM 12 | 19.08 (12.56, 29.34) | 18.45 (13.58, 25.56) | 0.706 |
| Variance GLCM 13 | 19.19 (12.94, 29.49) | 19.38 (13.57, 25.40) | 0.695 |

The data are presented as median with (1st quartile, 3rd quartile); ^a^, ×10^-3^; AFP, alpha fetoprotein; CEA, carcinoembryonic antigen; CA, carbohydrate antigen; N, non-enhanced phase; AP, arterial phase; PP, portal phase; DP, delayed phase; GLCM, gray-level cooccurrence matrix. **P*<0.05 with Mann-Whitney U test.

**Table A2** Interobserver agreement for CT morphological characteristics

| Characteristics | κ |
| --- | --- |
| Major location | 0.833 |
| Tumor range | 0.824 |
| Major orientation | 0.783 |
| Circumferential range | 0.786 |
| Infiltrative growth | 0.784 |
| Ulceration | 0.733 |
| Adjacent adipose tissue stains | 0.820 |
| Mucosal line status | 0.715 |
| Morphological type | 0.787 |
| Linitis plastic | 0.902 |
| Lymphadenectasis | 0.890 |

**Table A3** Interobserver agreement for CT value-related parameters

| Parameters | ICC | Parameters | ICC |
| --- | --- | --- | --- |
| N value mean | 0.802 | DP value mean | 0.941 |
| N value max | 0.737 | DP value max | 0.934 |
| N value min | 0.687 | DP value min | 0.872 |
| AP value mean | 0.941 | Δmean A-N | 0.935 |
| AP value max | 0.929 | Δmean P-N | 0.894 |
| AP value min | 0.886 | Δmean D-N | 0.916 |
| PP value mean | 0.917 | Δmean P-A | 0.893 |
| PP value max | 0.889 | Δmean D-A | 0.934 |
| PP value min | 0.856 | Δmean D-P | 0.855 |

ICC, intraclass correlation coefficient; N, non-enhanced phase; AP, arterial phase; PP, portal phase; DP, delayed phase.

**Table A4** Interobserver agreement for texture parameters

| Parameters | ICC | Parameters | ICC |
| --- | --- | --- | --- |
| Mean | 0.970 | Entropy GLCM 10 | 0.851 |
| Standard deviation | 0.849 | Entropy GLCM 11 | 0.857 |
| Max frequency | 0.988 | Entropy GLCM 12 | 0.843 |
| Mode | 0.933 | Entropy GLCM 13 | 0.845 |
| Minimum | 0.812 | Energy GLCM 10 | 0.846 |
| Maximum | 0.956 | Energy GLCM 11 | 0.866 |
| 5^th^ percentile | 0.907 | Energy GLCM 12 | 0.822 |
| 10^th^ percentile | 0.926 | Energy GLCM 13 | 0.853 |
| 25^th^ percentile | 0.950 | Inertia GLCM 10 | 0.782 |
| 50^th^ percentile | 0.969 | Inertia GLCM 11 | 0.870 |
| 75^th^ percentile | 0.982 | Inertia GLCM 12 | 0.845 |
| 90^th^ percentile | 0.989 | Inertia GLCM 13 | 0.775 |
| Skewness | 0.706 | Variance GLCM 10 | 0.871 |
| Kurtosis | 0.764 | Variance GLCM 11 | 0.862 |
| Entropy | 0.844 | Variance GLCM 12 | 0.864 |
| Histogram width | 0.859 | Variance GLCM 13 | 0.862 |

ICC, intraclass correlation coefficient; GLCM, gray-level cooccurrence matrix.
